# Comparison of the predictive value of intraoperative MRI and intraoperative histopathological examination for diagnostic stereotactic biopsy yield in patients with brain gliomas

**DOI:** 10.1016/j.bas.2026.106170

**Published:** 2026-07-15

**Authors:** Petrzelka, Sichova, Majovsky, Svoboda, Sedlak, Soukup, Komarc, Kazda, Netuka

**Affiliations:** a Department of Neurosurgery and Neurooncology, First Faculty of Medicine, Charles University and Military University Hospital, Prague, Czech Republic; b Institute of Biophysics and Informatics, First Faculty of Medicine, Charles University, Prague, Czech Republic; c Department of Methodology, Faculty of Physical Education and Sport, Charles University, Prague, Czech Republic; d Department of Pathology, Military University Hospital, Prague, Czech Republic; e Department of Radiodiagnostic, Military University Hospital, Prague, Czech Republic; f Department of Radiation Oncology, Masaryk Memorial Cancer Institute and Faculty of Medicine, Masaryk University, Brno, Czech Republic; g Department of Pathology, Charles University, First Faculty of Medicine and General University Hospital in Prague, Prague, Czech Republic; h The Fingerland Department of Pathology, Charles University, Faculty of Medicine in Hradec Králové and University Hospital, Hradec Kralove, Czech Republic

**Keywords:** Stereotactic biopsy, Gliomas, Intraoperative magnetic resonance imaging, Intraoperative histological examination, Neurooncology

## Abstract

Stereotactic biopsy remains the diagnostic gold standard for diffuse gliomas in patients unsuitable for surgical resection. Despite the high precision stereotactic techniques, intraoperative MRI (iMRI) and histological examination (iHE) are used to verify targeting accuracy and biopsy yield during surgery.

**Research question:**

This study aimed to compare the predictive value of iMRI and iHE for achieving diagnostic biopsy yield in frameless stereotactic biopsy of suspected diffuse glioma.

**Material and methods:**

We retrospectively analyzed patients who underwent frameless stereotactic biopsy using the VarioGuide system (BrainLab AG, Germany) at the MilitaryUniversityHospital Prague between April 2012 and February 2025. Patients with definitive diagnosis of diffuse glioma and intraoperative adjunct examinations during surgery were included. Sensitivity, specificity, positive predictive value, and negative predictive value of iMRI and iHE were evaluated. Secondary objectives included operative duration, overall survival, and comparison with CNS lymphoma biopsies

**Results:**

Definitive histology was diagnostic in 97.6% of 85 biopsies. Re-biopsy during the same procedure was required in 3.5% of patients, repeat biopsy under separate general anesthesia was necessary in one case. iMRI demonstrated higher sensitivity (96.4% vs. 68.7%) and negative predictive value (25.0% vs. 3.7%) compared to iHE. No significant difference in diagnostic yield was observed between biopsies performed with or without intraoperative adjuncts. Median overall survival in the high-grade glioma cohort was 4.0 months and associated with ECOG performance status.

**Discussion:**

Despite high accuracy of frameless stereotactic system, iMRI and iHE proved useful for intraoperative prediction of diagnostic biopsy yield. iMRI may additionally provide detection of possible perioperative complications. Poor survival outcomes reflected the unfavorable clinical status of this biopsy-only cohort.

## Highlights

• Stereotactic biopsy is the diagnostic gold standard for brain gliomas when resection is not feasible.
• Intraoperative MRI (iMRI) and intraoperative histology (iHE) are used to verify biopsy targeting and yield. Both techniques provide intraoperative feedback and allows to obtain additional bioptic samples, if necessary
• This study compares the predictive value of iMRI and iHE in achieving diagnostic biopsy yield in suspected diffuse gliomas.

## Abbreviation list:

MRI -: Magnetic Resonance Imaging
iMRI -: Intraoperative Magnetic Resonance Imaging
iHE –: intraoperative Histological Examination
WHO –: World Health Organization
IDH –: Isocitrate dehydrogenase
VG -: VarioGuide

## 1 Introduction

Diffuse gliomas are the most frequent malignant primary tumors of the adult CNS, with glioblastoma IDH-wildtype representing the most common and most aggressive subtype (Louis et al., 2021; van den Bent et al., 2023). Although tumor resection is mostly indicated, biopsy is preferred in situations where the lesion is radiologically uncertain, located in highly eloquent areas, or when patients with radiologically apparent high-grade glioma (HGG) are in poor clinical condition, unlikely to benefit from resection (Blakstad et al., 2023; Brown et al., 2022; Hertler et al., 2023; Louis et al., 2021). In such cases, obtaining tissue samples for histological and molecular diagnosis is necessary for possible following treatment.

For those, stereotactic biopsy remains the gold standard for obtaining representative tissue, offering high precision with relatively low morbidity. Frame-based stereotaxy has traditionally been valued for its accuracy and has been widely applied in both biopsies and functional neurosurgery. However, frameless systems have emerged as reliable alternatives, offering comparable accuracy and diagnostic yield while improving patient comfort and operational efficiency.

Despite careful planning of trajectory and target, biopsy specimens are not always diagnostic. Intraoperative histology (iHE) and intraoperative MRI (iMRI) with their increasing availability have therefore been implemented to confirm accurate targeting and sample adequacy (Brown et al., 2022; Buchalla et al., 2013; Budke et al., 2018; Dhawan et al., 2024; Hertler et al., 2023; Khatab et al., 2014; Zhang et al., 2018). Both methods provide immediate feedback and allow repeat sampling during the same procedure, if necessary, potentially improving diagnostic yield and reducing the need for additional interventions.

This study aimed to assess the predictive value of iMRI and iHE to confirm sufficient yield of biopsy, resulting in diagnostic definitive histology in patients with suspected diffuse gliomas. Identifying the more reliable intraoperative method may contribute to optimizing workflow, minimizing costs and length of surgery, while maintaining high diagnostic accuracy. As secondary objectives, we evaluated overall survival within this biopsy-only glioma cohort, analyzed the prognostic impact of ECOG performance status on patient outcomes, assessed the impact of intraoperative adjunct examinations on operative duration, and compared the predictive value of iMRI and iHE with our previously published results from CNS lymphoma biopsies.

## 2 Methods

### 2.1 Patients and data collection

We retrospectively analyzed data from patients who underwent stereotactic frameless brain biopsy using the VarioGuide system at the Department of Neurosurgery and Neurooncology, Military University Hospital Prague, performed between April 2012 and February 2025.

An initial list of all relevant surgeries, which operation record label include biopsy, frameless, stereotactic, VarioGuide or VG, was exported from the electronic hospital information system. This export of 453 patients included patient names, birth numbers, ages, and dates of surgery. Data were manually reviewed to identify patients who met the inclusion criteria of VarioGuide-guided biopsy, use of both iMRI and iHE during the surgery, and definitive histological diagnosis of diffuse glioma. Additional data, such as tumor localization, operative duration and ECOG performance score (Table 1) during admission were collected. Histopathological grading was performed according to the WHO CNS 2021 classification. For biopsies performed before the 2021 classification update, the diagnoses were reclassified according to the new criteria based on their original histological reports. Patient records were further reviewed, and subsequent therapy information, when available, was recorded. Survival status and date of death were verified through the National Health Insurance registry.

**Table 1:** ECOG performance status scale.

ECOG Grade	
0	Fully active, able to carry on all pre-disease performance without restriction
1	Restricted in physically strenuous activity but ambulatory and able to carry out work of a light or sedentary nature, e.g., light house work, office work
2	Ambulatory and capable of all selfcare but unable to carry out any work activities; up and about more than 50% of waking hours
3	Capable of only limited selfcare; confined to bed or chair more than 50% of waking hours
4	Completely disabled; cannot carry on any selfcare; totally confined to bed or chair
5	Dead

Additional analysis was performed to compare the main cohort undergoing both intraoperative adjunct examinations with a subgroup of glioma biopsies initially excluded from the study cohort, in which only one or neither intraoperative adjunct was performed to find possible impact on biopsy yield and overall length of surgery. Findings of intraoperative examinations predictive value were also compared with results evaluating predictive value of iHE and iMRI in stereotactic biopsies of suspected brain lymphoma in our department, published by Májovský et al. (Majovsky et al., 2024).

### 2.2 VarioGuide

**Fig. 1:**
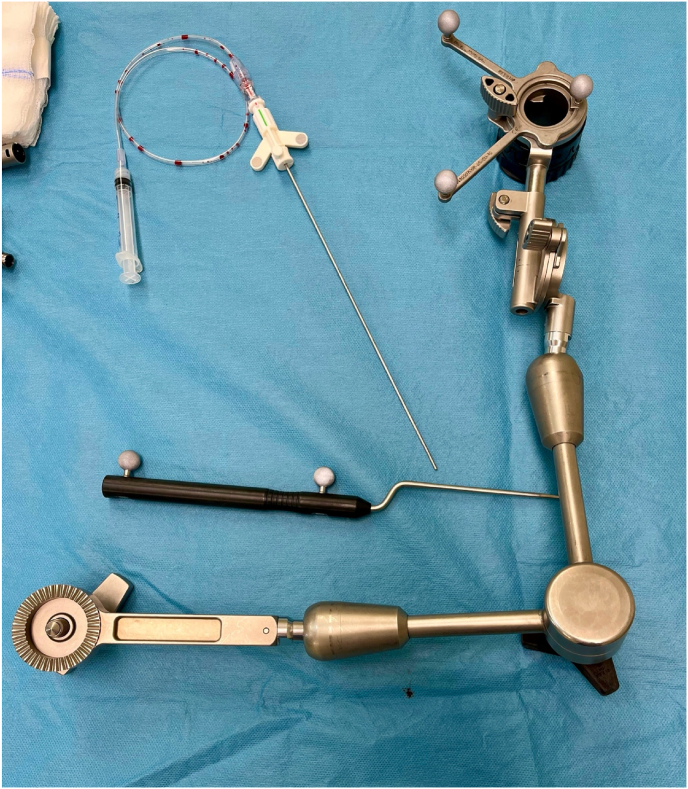
VarioGuide setup.

The frameless stereotactic device used in this study was the VarioGuide 1.0 (VG) by BrainLab AG, Germany (Fig. 1). The system consists of a reference array and a VG arm connected in our setup, to a Mayfield three-point head clamp. The VG's articulated three-joint arm allows precise adjustment of the instrument holder at the arm's distal end. The positions the VG and patient and biopsy needle are live-tracked by infrared (IR) cameras using the reference array on the patient and on the VG (Almeida et al., 2018; Bradac et al., 2017; Buchalla et al., 2013).

### 2.3 Surgery

The trajectory and target were planned preoperatively by the surgeon using the navigation sequences, typically 3D T1w contrast enhanced sequences in case of enhancing tumors and 3D T2w sequences for non-enhancing tumors, in both cases with 1 mm isotropic resolution to ensure the entire trajectory remained within the brain tissue or tumor, avoiding vascular structures. Most prominent targets, i.e. enhancing part of the tumor, avoiding necrotic core, were selected. All trajectories were planned using BrainLab planning software.

**Fig. 2:**
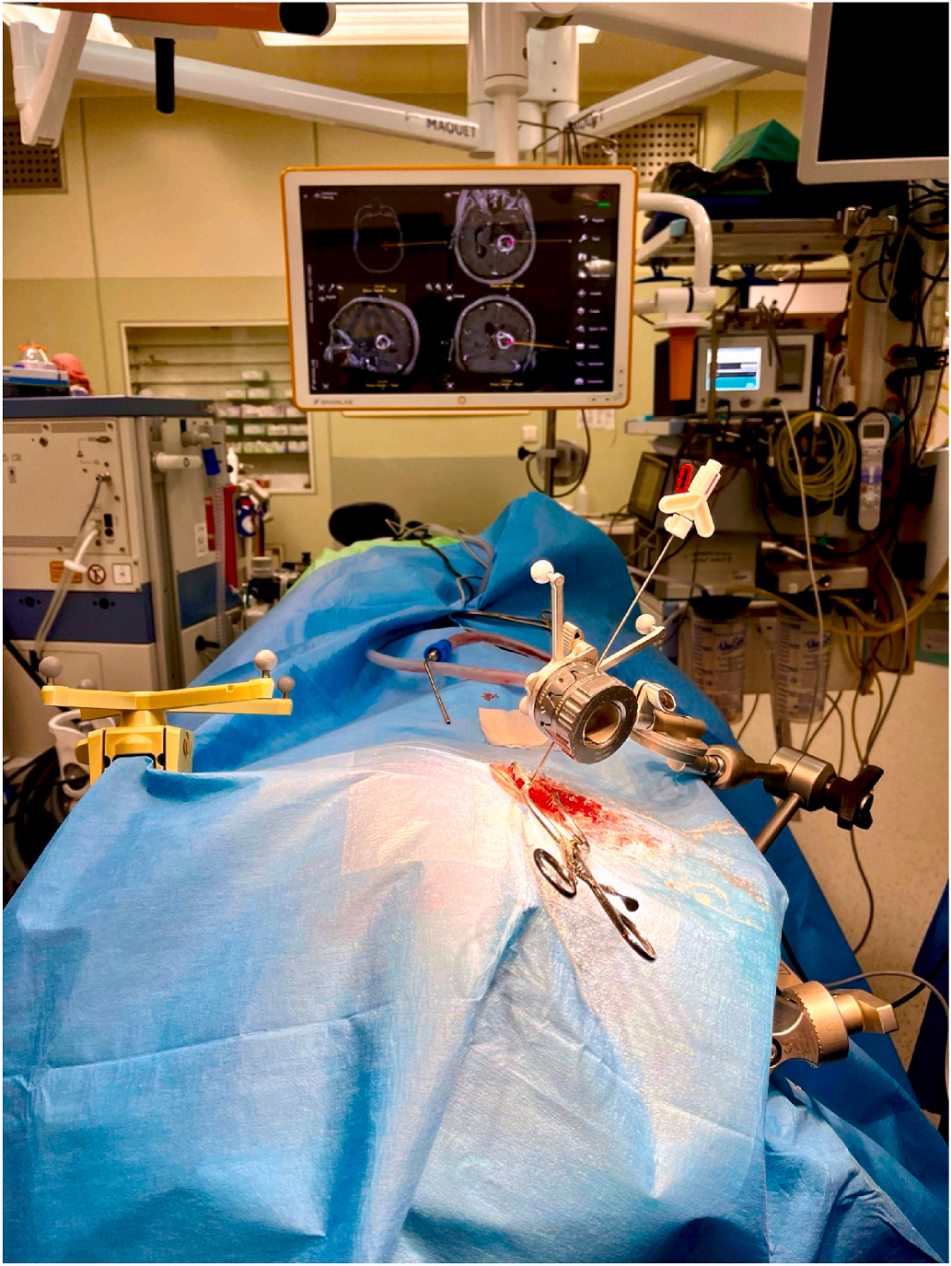
OR, VarioGuide, needle placed in target location.

Surgeries were performed under general anesthesia. After placement of the Mayfield head clamp, neuronavigation was registered. Following standard sterile preparation and draping, a small burr hole was created at the planned entry point. The VarioGuide system was then mounted, and the needle trajectory through the burr hole was verified to ensure unobstructed needle entry (Fig. 2).

### 2.4 Intraoperative histological examination (iHE)

After samples were collected, one to three samples were submitted to the pathology department for intraoperative histological examination, while further samples were obtained for definitive histology. Samples were prepared according to the department's internal guidelines: the specimen was placed on a 25 mm cryostat chuck using frozen section gel and transferred to the cryotome, where it was frozen at −44 °C. At least three tissue sections, each 4 μm thick, were cut and mounted on glass slides. The slides were stained with Weigert's hematoxylin for 2 min followed by eosin for 30 s, then mounted and sent to the pathologist for diagnostic assessment. Samples for definitive histology were fixed in 4% formaldehyde, paraffin-embedded, and sectioned according to the department's routine protocol.

All the frozen samples were assessed by a dedicated neuropathologist with more than 10 years experience. Positive iHE findings were defined as confirmation of neoplastic glioma tissue by the neuropathologist. Negative or inconclusive findings included samples containing normal brain tissue, nonspecific changes such as reactive gliosis without unequivocal tumor cells, or specimens in which the presence of tumor could not be confirmed due to necrosis, hemorrhage, or insufficient tissue quality.

### 2.5 Intraoperative magnetic resonance imaging (iMRI)

Following the surgical procedure, iMRI was performed under general anesthesia using a 3T GE 750w system (GE Healthcare). If the iMRI confirmed accurate biopsy localization and revealed no surgical complications, the procedure was concluded. If complications or inaccurate targeting were detected, we would resume the surgery to obtain additional samples or to address the identified issues.

Positive iMRI findings were defined as confirmation that the biopsy needle accurately reached the intended target lesion. Negative or inconclusive findings were defined as needle deviation from the planned target or biopsy localization limited to the lesion periphery on intraoperative MRI. In cases of inaccurate targeting on iMRI, repeat navigation registration was performed, a new trajectory was planned, and additional biopsy samples were obtained.

## 3 Statistical analysis

Descriptive statistics were used to summarize patient demographics, tumor localization, and surgical outcomes. Categorical variables are reported as frequencies and percentages.

Overall survival (OS) was defined as the interval between biopsy and death, with surviving patients censored on May 1, 2025. Survival probabilities were estimated using the Kaplan-Meier method, which accounts for censored observations. To compare survival between ECOG subgroups, the log-rank test was applied because it directly tests differences in Kaplan-Meier survival curves.

To provide a more comprehensive description of survival beyond median OS, we also calculated the Restricted Mean Survival Time (RMST) with τ set at 24 months, as this method captures the average survival time up to a fixed horizon and is less sensitive to small numbers of long-term survivors.

To assess intraoperative diagnostic accuracy, we calculated sensitivity, specificity, positive predictive value (PPV), negative predictive value (NPV), and area under the receiver operating characteristic curve (AUC) for iHE and iMRI, as these measures quantify the reliability of intraoperative findings compared with definitive histology.

All analyses were conducted using R (version 4.4.2), SPSS (version 25) and Python 3.13.

## 4 Results

A total of 453 surgeries were initially identified in HIS, out of which 85 met the inclusion criteria and were further analyzed. (Table 2)

**Table 2:** Patients characteristics.

	n = 85
**Age**	
Median (range)	63.5 (22-84)
**Sex**	
Male	47 (55.3%)
Female	38 (44.7%)
**ECOG**	
ECOG 0	14 (16.5%)
ECOG 1	35 (41.2%)
ECOG 2	23 (27.1%)
ECOG 3	10 (11.8%)
ECOG 4	3 (3.5%)
**Localization**	
Frontal lobe	30 (35.3%)
Parietal lobe	23 (27.1%)
Temporal lobe	17 (20.0%)
Occipital lobe	5 (5.9%)
Deep structures	10 (11.8%)
**IDH status**	
Wild-type	67 (78.8%)
IDH-mut	13 (15.3%)
Unavailable	5 (5.9%)
**Definitive histology**	
Grade 2	8 (9.4%)
Grade 3-4	75 (88.2%)
Failed biopsy	2 (2.4%)

Definitive histology confirmed high-grade glioma (HGG) in 88.2% (n = 75), low-grade glioma (LGG) in 9.4% (n = 8), but was not diagnostic in 2.4% (n = 2), resulting in an overall biopsy yield rate of 97.6% (95% CI 91.7%–99.7%, n = 83). Necessity of re-biopsy after iMRI, during single surgery, was required in 3.5% of cases (n = 3), and necessity for further biopsy in another general anesthesia occurred in 1.2% (n = 1).

In our series, none of the 85 biopsies were associated with perioperative or postoperative complications.

### 4.1 Predictive value of iMRI and iHE

Positive predictive value (PPV) of iMRI and iHE was not significantly different (98.8%, 80/81, CI [93.2–100%] vs. 98.3%, 57/58, [CL 90.8–100%]; Fisher's exact test, p = 1.00).

Negative predictive value (NPV) was higher for iMRI, but did not show statistical significance (25.00%, 1/4, CI [0.6–75.9%] vs. 3.7%, 1/27, CI [0.1–19.0%]; Fisher's exact test, p = 0.25).

Sensitivity of iMRI was significantly higher (96.4%, CI [89.8–99.2%] vs. 68.7%, CI [57.6–78.4%] for iHE; Fisher's exact test, p < 0.001) (Table 3).

**Table 3:** Predictive value of iMRI and iHE TP – True Positive, FP – False Positive, TN – True Negative, FN – False Negative, PPV – Positive Predictive Value, NPV – Negative Predictive Value.

	iHE	iMRI
**TP**	57	80
**FP**	1	1
**TN**	1	1
**FN**	26	3
**PPV**	98.28% (57/58), CI [90.76–99.96%]	98.77% (80/81), CI [93.24–99.97%]
**NPV**	3.70% (1/27), CI [0.09–18.96%]	25.00% (1/4), CI [0.63–75.88%]
**Sensitivity**	68.67% (57/83), CI [57.62–78.41%]	96.39% (80/83), CI [89.77–99.22%]
**Specificity**	50.00% (1/2), CI [1.26–98.74%]	50.00% (1/2), CI [1.26–98.74%]

**Fig. 3:**
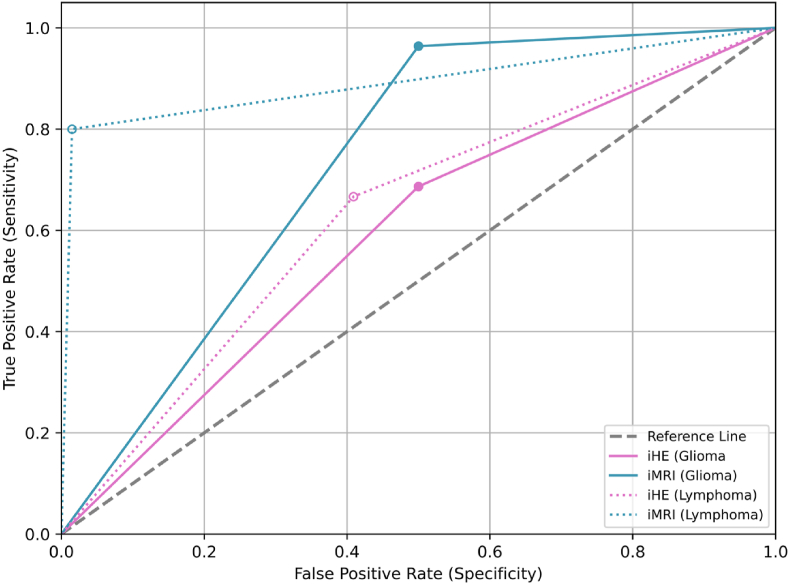
ROC Comparison: iHE and iMRI in Glioma vs. Lymphomas group.

Specificity for both iMRI and iHE was 50.0% (1/2), CI [1.3–98.7%], though limited by the small number of non-diagnostic cases (n = 2). The AUC of ROC curves, was calculated for iHE 0.6 and 0.7 for iMRI (Fig. 3).

### 4.2 Comparison of biopsy yield among iMRI, iHE-only, and no-intraoperative adjunct groups and the impact of iMRI on length of surgery

Additional biopsies of diffuse brain gliomas performed with iMRI only (n = 125), iHE only (n = 6), or without intraoperative adjunct examinations (n = 39) were evaluated to assess the impact on diagnostic yield and operative time. Compared with the main cohort undergoing both iMRI and iHE, no statistically significant difference in diagnostic yield was observed for iMRI-only biopsies (92.5%, 119/125; 95% CI [0.10–2.33]; p = 0.13), iHE-only biopsies (83.3%, 5/6; CI [0.01–1.45]; p = 0.19), or biopsies performed without intraoperative examinations (94.9%, 37/39; CI [0.07–2.92]; p = 0.39).

The use of iMRI was associated with longer operative duration compared to procedures performed without iMRI (n = 210, median 56.5 min, range 28–120min vs. n = 45, median 35 min, range 15–139min; p < 0.001, Mann–Whitney *U* test).

### 4.3 Survival

**Fig. 4:**
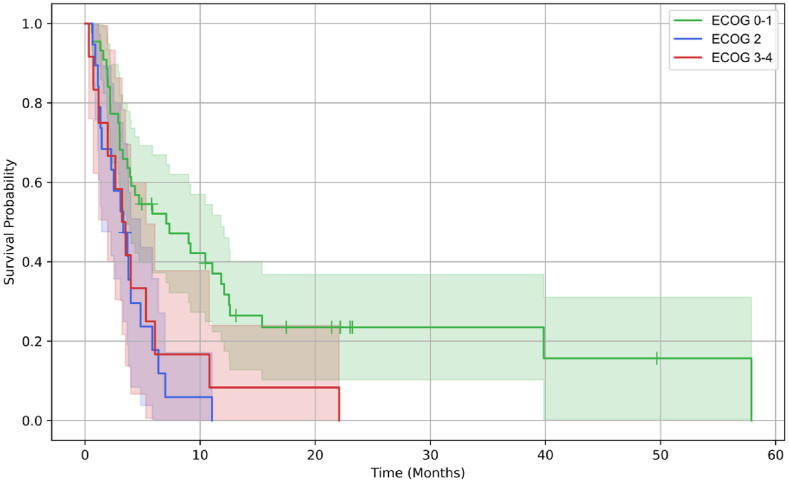
Kaplan-Meier survival analysis of HGG group.

Median overall survival (OS), calculated using the Kaplan-Meier method (Fig. 4), among the 75 patients in the HGG cohort (censored at May 1, 2025 for the 11 alive patients) was 4.0 months (95% CI: 2.9-5.0 months). When stratified by combined ECOG groups, median OS for ECOG 0-1 (n = 44) was 7.0 months, (95% CI: 1.4-12.7), for ECOG 2 (n = 19) 3.3 months (95% CI: 1.7-4.9), for ECOG 3-4 (n = 12) 3.2 months (95% CI: 1.7-4.7).

For the remaining ten patients, eight diagnosed with low-grade glioma and two without a definitive histological diagnosis, overall survival was not calculated due to the small sample size, which would not provide representative outcomes.

For a more comprehensive view on survival, Restricted Mean Survival Time (RMST) was calculated for the group of all HGG (n = 75), using τ = 24 months, as 7.69 months (95% CI: 5.91-9.46 months). For the ECOG 0-1 subgroup, RMST was 10.08 months (95% CI: 7.49-12.68 months), for the ECOG 2 subgroup 3.69 months (95% CI: 2.48-4.90 months), and for ECOG 3-4 5.15 months (95% CI: 1.87-8.43 months).

These findings were further supported by the log-rank test, with an overall comparison across ECOG groups yielding a χ^2^ of 13.39 (df = 2, p ≤ 0.001), indicating significant heterogeneity. Pairwise comparisons revealed a significant difference between ECOG 0-1 and ECOG 2 (HR 2.76, 95% CI: 1.50-5.11; χ^2^ = 11.35, df = 1, p ≤ 0.001) and between ECOG 0-1 and ECOG 3-4 (HR 2.28, 95% CI: 1.16-4.46; χ^2^ = 5.46, df = 1, p = 0.020), while ECOG 2 versus ECOG 3-4 (HR 1.20, 95% CI: 0.56-2.56; χ^2^ = 0.23, df = 1, p = 0.631) showed no significant difference, aligning with the Cox model's nuanced hazard estimates.

### 4.4 Following therapy of HGG group

After the definitive histology and diagnosis was made, treatment was recommended according to our neuro-oncology team and patients were referred to oncology department based on their place of residence.

Within the HGG group, 46 patients received concomitant chemoradiotherapy (CHRT) (ECOG 0-1 n = 29, ECOG 2 n = 12, ECOG 3-4 n = 5), one patient was treated with chemotherapy alone (ECOG 0-1), and 16 patients received palliative radiotherapy (RT) (ECOG 0-1 n = 8, ECOG 2 n = 3, ECOG 3-4 n = 5). Additionally, four patients underwent surgical resection following the biopsy (ECOG 0-1 n = 3, ECOG 2 n = 1). In two cases, treatment information was not available as patients did not attend postoperative control. We opted for monitoring through MRI for four patients (ECOG 0-1 n = 1, ECOG 2 n = 2, ECOG 3-4 n = 1).

One patient, whose diagnosis was based on iHE and radiological appearance, even though the definitive histology was inconclusive; however, with positive iMRI and iHE, was treated according to the iHE with a diagnosis of high-grade glioma (HGG). As a result, this patient received palliative radiation therapy (RT). In second case of failed biopsy, details regarding the therapeutic approach remain unavailable because of the patient died in the same month of undergoing the biopsy.

## 5 Discussion

Our findings showed greater predictive value of iMRI compared to iHE in confirming sufficient biopsy yield, with higher sensitivity (96.4% vs. 68.7%) and a higher negative predictive value (25.0% vs. 3.7%), while both methods were evaluated under identical procedural conditions within the same biopsy cohort. An additional advantage of iMRI is its ability to detect intraoperative complications, such as hemorrhage, providing a time-saving feature that may influence outcomes by allowing immediate surgical decision-making. High sensitivity and negative predictive value of iMRI may also reflect the high accuracy of the VG, as previously demonstrated (Almeida et al., 2018; Bradac et al., 2017; Buchalla et al., 2013; Budke et al., 2018; Ladisich et al., 2021; Ringel et al., 2009; Ungar et al., 2022; Viozzi et al., 2024; Vychopen et al., 2022; Zhang et al., 2018).

The diagnostic yield in our cohort (97.6%) is consistent with previously reported results for frameless stereotactic biopsies. Bradáč et al. achieved a yield of 92.5% in 27 VG-guided biopsies (Bradac et al., 2017), while a systematic review by Khatab et al. reported a mean yield of 93.8% across 16 studies (Khatab et al., 2014).

The lower precision of intraoperative histology stems largely from its reliance on small frozen sections, which are known to have technical limitations. Additionally, iHE offers a “one time snapshot” of the tissue sampled during the biopsy procedure, out of the real time anatomical context inherent to iMRI procedure. Thus, it is highly dependent on the first successful targeting of the desired brain tissue area.

### 5.1 Failed biopsy

Out of 85 definitive biopsies, two biopsies did not result as diagnostic and were inefficient (1,7%). Although these two patients did not strictly meet our inclusion criteria, their diagnoses were classified as HGG based on their characteristic radiological appearance. In the first of those, neither iHE nor iMRI suggested adequate biopsy, and were classified as negative. In the second case, definitive histology was not diagnostic, despite both iMRI and iHE classifying as positive, as the pathologist couldn't discriminate minimal infiltration by glioma and reactive gliosis of the surrounding parenchyma due to the small amount of material obtained from definitive histology. Despite the nonconclusive definitive histology, the patient received treatment according to result of diagnostic positive iHE and MR characteristics, suggesting HGG.

An insufficient number of samples to accurately represent the tumor may contribute to negative iHE findings or inconclusive definitive histology. Meta-analyses published by Dhawan et al. (2024) have shown that the number of samples obtained during stereotactic biopsy has little influence on diagnostic yield, which remains consistently high. However, morbidity increases with higher sample counts. These findings suggest that obtaining a moderate number of samples, typically 3-6, provides an optimal balance. This sampling strategy corresponds to the practice followed in our department.

### 5.2 Survival

The median overall survival for the HGG group of 75 patients was 4.0 months, lower than the reported median OS for glioblastoma in clinical trial populations, which range from 14 to 17 months, but consistent with the poor prognosis typically observed in biopsy-only glioma cohorts. (Blakstad et al., 2023; Brown et al., 2022; Hertler et al., 2023; Livermore et al., 2014; Louis et al., 2021; van den Bent et al., 2023). ECOG performance status showed clear prognostic significance. Patients with ECOG 0-1 (6.86 months) had the longest survival, while those with ECOG 2 and 3-4 had significantly shorter outcomes (3.20 and 3.22 months). No clear difference in OS between ECOG groups 2 and 3-4 may be partly explained by the smaller number of patients in the latter group. Overall, these results indicate that once performance status worsens beyond ECOG 1, survival expectations are markedly reduced.

When interpreting overall survival in our cohort it should be noted that patients with suspected high-grade glioma who underwent biopsy rather than resection were, in the judgment of the neuro-oncology team, generally in poorer overall condition or presented with severe neurological deficits related to tumor size. In some cases, the risk of causing additional neurological deterioration with resection was considered high, with likely worsening of quality of life or without expected functional benefit. For these reasons, resection was considered unlikely to improve prognosis or functional outcome, and biopsy was performed to confirm the diagnosis and allow timely initiation of oncological therapy. However, surgical resection alone does not necessarily translate into prolonged survival, as factors such as extent of resection, perioperative complications must also be considered. Primary therapy following biopsy has remained chemoradiotherapy. Further information regarding oncological treatment was unavailable in most cases, as patients were referred to regional oncology centers according to their place of residence.

### 5.3 Impact of intraoperative adjuncts on biopsy yield and operative duration

In the additional cohort analysis, no statistically significant difference in diagnostic yield was observed between biopsies performed with both iMRI and iHE, iMRI alone, iHE alone, or without intraoperative adjunct examinations. These findings suggest that frameless stereotactic biopsy using the VarioGuide system provides high diagnostic accuracy. In three cases, iMRI showed inaccurate targeting, enabling re-biopsy under the same general anesthesia after planning a new biopsy trajectory, resulting in diagnostic definitive histology despite inconclusive iHE findings in one case. No re-biopsy was performed in cases with inconclusive iHE findings if iMRI confirmed accurate targeting, as the results of both examinations were usually available at approximately the same time, with the decision on re-biopsy guided primarily by the iMRI findings. Additional analysis of this cohort also provided insight into operative duration, which was significantly longer in procedures utilizing intraoperative MRI. In our setup, however, the prolonged operative time reflected not only MRI acquisition itself, but also the need for patient and MRI suite preparation, while the scanner continued to serve routine diagnostic examinations during the procedure.

### 5.4 Comparison with Lymphoma biopsies

In comparison to predictive value of iMRi and iHE to biopsy yield in VG biopsy of CNS lymphomas in our department, published by Májovský et al. (Majovsky et al., 2024), we observed that iMRI provided more consistent accuracy across both tumor types. In gliomas, sensitivity reached 96.4%, while in lymphomas it was slightly lower at 80.0%. Specificity was also high in lymphoma group (98.5%), confirming iMRI as a reliable method for verifying correct targeting.

For iHE, the negative predictive value was higher in lymphomas (96.3% vs. 3.7%), suggesting that in lymphomas biopsies, iHE can serve as a more trustable method for ruling out false negative samples. This can be due to the different pattern of parenchymal involvement in lymphomas that tend to infiltrate around the vessels, including small arterioles and capillaries. Therefore, brain parenchyma involvement can be more diffuse and include also discrete foci in the areas not yet recognizable on iMRI. On the other hand, this may also explain lower overall sensitivity of both methods when dealing with lymphoma. Since even the low-grade gliomas with limited cellularity tend to form a homogeneous area of involvement well identifiable on MRI, it would not be surprising to skip scattered foci of perivascular lymphoma during the biopsy. Additionally, perivascular involvement by lymphoma characteristically leads to increased vessel permeability and contrast-enhancement compared to gliomas (Lee et al., 2019), that may result in mismatch between contrast enhancing regions and local tumor cell density, potentially contributing to false-negative biopsy samples despite radiologically apparent lesions.

## 6 Conclusion

In this study, we evaluated the role of intraoperative MRI and intraoperative histology during frameless stereotactic biopsies of diffuse gliomas. Both methods proved useful, but iMRI showed higher sensitivity and negative predictive value compared to iHE in cases where both intraoperative adjunct examinations were performed. iMRI also provided information about possible perioperative complications, which may help the surgeon react immediately. Although iHE is faster and less costly, iMRI provided greater reliability and safety, making it a valuable complement when available, despite prolonged operative time.

Nevertheless, comparison with our previously published CNS lymphoma biopsy cohort suggests that the predictive value of iMRI and iHE may differ between tumor types, supporting a role of iHE in selected cases.

Our findings also support the prognostic importance of ECOG performance status. Patients with good performance (ECOG 0-1) had longer survival, while those with ECOG ≥2 had markedly shorter outcomes. Overall survival in the biopsy-only glioma group remained poor, reflecting the fact that biopsy is often chosen for patients in worse condition or with tumors not suitable for resection.

Our results suggest that combining iMRI and iHE can increase diagnostic confidence and patient safety in stereotactic biopsies. However, the relatively small sample size represents a limitation of this study. Further studies with larger cohorts are needed to confirm these findings and define the optimal indications for intraoperative imaging. Additionally, the retrospective single-center design may introduce selection bias, and some mechanistic explanations discussed in this study should therefore be interpreted as hypotheses, rather than definitive conclusions.

## 7 Strengths and limitations

The main strength of this study is the direct comparison of iMRI and iHE within the same frameless stereotactic biopsy surgery under identical conditions. Additional strengths include evaluation of operative duration, overall survival, and comparison with our previously published CNS lymphoma cohort. Limitations include the retrospective single-center design, relatively small sample size, and the low number of non-diagnostic biopsies.

## Ethics approval and consent to participate

This study was reviewed and approved by the institutional ethics committee with approval number 108/20-35/2025.

## Funding

This research was supported by 10.13039/501100003243Ministry of Health of the Czech Republic in cooperation with the Czech 10.13039/100018696Health Council under project no. NW25J-08-00023, institutional support MO1012 by 10.13039/501100014911Ministry of Defence of the Czech Republic, 10.13039/100007397Charles University Grant Agency 10.13039/100007543GA UK No. 222623 and Cooperatio Neuroscience by 10.13039/100007397Charles University. The funders of the study had no role in the study design nor the collection, analysis, and interpretation of data, writing of the report, or decision to submit the manuscript for publication.

## Competing interests

The authors declare that they have no competing interests.
